# 3,3,6,6-Tetra­methyl-9-(2-nitro­phen­yl)-3,4,6,7-tetra­hydro-2*H*-xanthene-1,8(5*H*,9*H*)-dione

**DOI:** 10.1107/S1600536810029211

**Published:** 2010-07-31

**Authors:** Yingming Mo, Hong-Jun Zang, Bo-Wen Cheng

**Affiliations:** a Department of Enviromental and Chemistry Engineering, Tianjin Polytechnic University, State Key Laboratory of Hollow Fiber Membrane Materials and Processes, Tianjin 300160, People’s Republic of China

## Abstract

In the title compound, C_23_H_25_NO_5_, the pyran ring adopts a flattened boat conformation, while the two cyclo­hexenone rings are in envelope conformations. The 3-nitro­phenyl ring is almost perpendicular to the pyran ring, making a dihedral angle of 87.1 (3)°.

## Related literature

For the use of xanthenes as dyes and fluorescent materials for visualization of biomolecules and in laser technologies, see: Menchen *et al.* (2003 ▶); Banerjee & Mukherjee (1981 ▶). They can be converted by oxidation into xanthylium salts, which are also effective as dyes and fluorescent materials, see: Nogradi (2003 ▶); Kamel & Shoeb (1964 ▶). For the biological and pharmaceutical properties of xanthenes, see: Hideo (1981 ▶); Lambert *et al.* (1997 ▶); Poupelin *et al.* (1978 ▶).
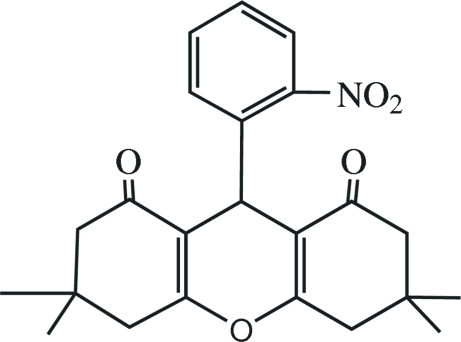


## Experimental

### 

#### Crystal data

C_23_H_25_NO_5_
*M* *_r_* = 395.44
Orthorhombic, 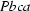

*a* = 12.199 (2) Å
*b* = 10.510 (2) Å
*c* = 32.484 (7) Å
*V* = 4164.9 (14) Å^3^
*Z* = 8
Mo *K*α radiation
μ = 0.09 mm^−1^
*T* = 113 K
0.20 × 0.16 × 0.10 mm

#### Data collection

Rigaku Saturn CCD area-detector diffractometer
Absorption correction: multi-scan (*CrystalClear*; Rigaku, 2002 ▶) *T*_min_ = 0.983, *T*_max_ = 0.991
21559 measured reflections
3670 independent reflections
3242 reflections with *I* > 2σ(*I*)
*R*_int_ = 0.053

#### Refinement

*R*[*F* ^2^ > 2σ(*F* ^2^)] = 0.063
*wR*(*F* ^2^) = 0.144
*S* = 1.16
3670 reflections
267 parameters
H-atom parameters constrained
Δρ_max_ = 0.22 e Å^−3^
Δρ_min_ = −0.24 e Å^−3^

### 

Data collection: *CrystalClear* (Rigaku, 2002 ▶); cell refinement: *CrystalClear*; data reduction: *CrystalClear*; program(s) used to solve structure: *SHELXS97* (Sheldrick, 2008 ▶); program(s) used to refine structure: *SHELXS97* (Sheldrick, 2008 ▶); molecular graphics: *SHELXTL* (Sheldrick, 2008 ▶); software used to prepare material for publication: *SHELXTL*.

## Supplementary Material

Crystal structure: contains datablocks I, publication_text. DOI: 10.1107/S1600536810029211/jh2186sup1.cif

Structure factors: contains datablocks I. DOI: 10.1107/S1600536810029211/jh2186Isup2.hkl

Additional supplementary materials:  crystallographic information; 3D view; checkCIF report

## Figures and Tables

**Fig. 1.**
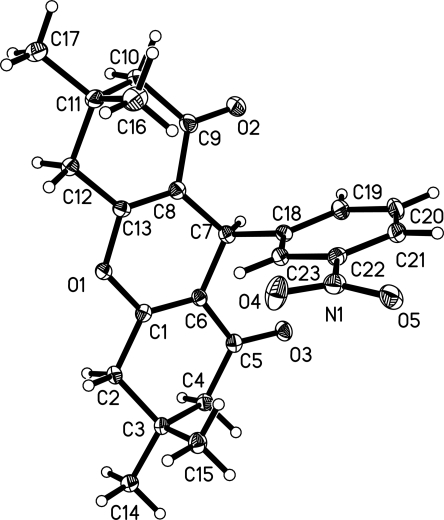
A view of the Structure of (I), Showing the atom-numbering scheme. Dispacement ellipsoids are drawn at the 30% probability level.

**Fig. 2.**
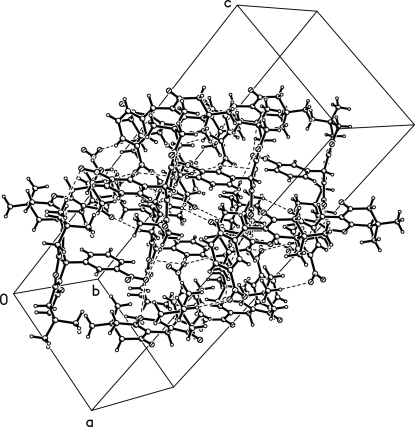
A packing diagram of the title molecule.

## supplementary crystallographic information

### Comment

Xanthenes are an important class of organic compounds that find use as dyes, fluorescent materials for visualization of biomolecules and in laser technologies, due to their useful spectroscopic properties (Menchen *et al.*, 2003; Banerjee & Mukherjee, 1981). Oxidation of these compounds can be converted to the corresponding xanthylium salts, which are also effective as dyes and fluorescent materials (Nogradi, 2003; Kamel & Shoeb, 1964). Xanthenes have also received considerable attention from many pharmaceuticals and organic chemists, actually because of the broad spectrum of their biological and pharmaceutical properties such as agricultural bactericide effects (Hideo, 1981), photodynamic therapy, anti-inflammatory activities (Poupelin *et al.*, 1978) and antiviral effects (Lambert *et al.*, 1997). In view of the importance of the title compound,(I), we report herein its crystal structure.

The pyran ring of the title molecule (Fig. 1) adopts a flattened boat conformation. The two cyclohexenone rings adope envelope conformation with atom C3 and C11 at the flap. The 3-nitrophenyl ring and the planar part of the pyran ring (C1/C6/C8/C13) are nearly perpendicular to each other, with a dihedral angle of 87.1 (3)°.

### Experimental

A mixture of 2-nitrobenzaldehyde (212 mg, 2 mmol), dimedone (560 mg, 4 mmol), *p*-TSA (2 mg, 5 mol%), 4 ml of MeOH containing 2 ml of water was heated to 50 ° C in an atmosphere of argon for about 20 min. After completion of the reaction (as indicated by TLC), the reaction mixture was poured into crushed ice and stirred for about 1 h.The solid separated was filtered through a sintered funnel under suction, washed with ice-cold water (30 ml) and then recrystallized from hot ethanol to afford the product (0.213 g, 80%). A single-crystal was obtained by slow evaporation of a EtOH solution.

### Refinement

The H atoms bonded to C atoms were included in the refinement in the riding model approximation, with C–H = 0.93–0.97 Å and *U*_iso_ (H) = 1.2 *U*_eq_ (C atom). For the H atoms attached to C atoms of methyl groups, their *U*_iso_(H) =1.5*U*_eq_(C).

### Crystal data

**Table:**

C_23_H_25_NO_5_	*D*_x_ = 1.261 Mg m^−^^3^
*M**_r_* = 395.44	Mo *K*α radiation, λ = 0.71073 Å
Orthorhombic, *P**b**c**a*	Cell parameters from 9106 reflections
*a* = 12.199 (2) Å	θ = 1.9–28.1°
*b* = 10.510 (2) Å	µ = 0.09 mm^−^^1^
*c* = 32.484 (7) Å	*T* = 113 K
*V* = 4164.9 (14) Å^3^	Prism, white
*Z* = 8	0.20 × 0.16 × 0.10 mm
*F*(000) = 1680	

### Data collection

**Table:**

Rigaku Saturn CCD area-detector diffractometer	3670 independent reflections
Radiation source: rotating anode	3242 reflections with *I* > 2σ(*I*)
confocal	*R*_int_ = 0.053
Detector resolution: 7.31 pixels mm^-1^	θ_max_ = 25.0°, θ_min_ = 2.5°
ω and φ scans	*h* = −14→14
Absorption correction: multi-scan (*CrystalClear*; Rigaku, 2002)	*k* = −10→12
*T*_min_ = 0.983, *T*_max_ = 0.991	*l* = −38→36
21559 measured reflections	

### Refinement

**Table:**

Refinement on *F*^2^	Secondary atom site location: difference Fourier map
Least-squares matrix: full	Hydrogen site location: inferred from neighbouring sites
*R*[*F*^2^ > 2σ(*F*^2^)] = 0.063	H-atom parameters constrained
*wR*(*F*^2^) = 0.144	*w* = 1/[σ^2^(*F*_o_^2^) + (0.0574*P*)^2^ + 1.5964*P*] where *P* = (*F*_o_^2^ + 2*F*_c_^2^)/3
*S* = 1.16	(Δ/σ)_max_ = 0.001
3670 reflections	Δρ_max_ = 0.22 e Å^−^^3^
267 parameters	Δρ_min_ = −0.24 e Å^−^^3^
0 restraints	Extinction correction: *SHELXL*, Fc^*^=kFc[1+0.001xFc^2^λ^3^/sin(2θ)]^-1/4^
Primary atom site location: structure-invariant direct methods	Extinction coefficient: 0.0098 (8)

### Special details

**Table:**

Geometry. All e.s.d.'s (except the e.s.d. in the dihedral angle between two l.s. planes) are estimated using the full covariance matrix. The cell e.s.d.'s are taken into account individually in the estimation of e.s.d.'s in distances, angles and torsion angles; correlations between e.s.d.'s in cell parameters are only used when they are defined by crystal symmetry. An approximate (isotropic) treatment of cell e.s.d.'s is used for estimating e.s.d.'s involving l.s. planes.
Refinement. Refinement of *F*^2^ against ALL reflections. The weighted *R*-factor *wR* and goodness of fit *S* are based on *F*^2^, conventional *R*-factors *R* are based on *F*, with *F* set to zero for negative *F*^2^. The threshold expression of *F*^2^ > σ(*F*^2^) is used only for calculating *R*-factors(gt) *etc*. and is not relevant to the choice of reflections for refinement. *R*-factors based on *F*^2^ are statistically about twice as large as those based on *F*, and *R*- factors based on ALL data will be even larger.

### Fractional atomic coordinates and isotropic or equivalent isotropic displacement parameters (Å^2^)

**Table:**

	*x*	*y*	*z*	*U*_iso_*/*U*_eq_	
O1	0.54601 (12)	0.17933 (14)	0.36579 (4)	0.0237 (4)	
O2	0.25719 (14)	0.45727 (16)	0.40103 (5)	0.0387 (5)	
O3	0.49229 (13)	0.50696 (15)	0.26800 (4)	0.0306 (4)	
O4	0.77268 (15)	0.56112 (18)	0.42780 (6)	0.0474 (5)	
O5	0.78206 (14)	0.76618 (17)	0.42061 (5)	0.0400 (5)	
N1	0.73522 (16)	0.66330 (19)	0.41615 (5)	0.0299 (5)	
C1	0.56412 (18)	0.2415 (2)	0.32907 (6)	0.0219 (5)	
C2	0.64378 (18)	0.1708 (2)	0.30297 (6)	0.0227 (5)	
H2A	0.6054	0.0996	0.2892	0.027*	
H2B	0.7015	0.1340	0.3208	0.027*	
C3	0.69762 (18)	0.2553 (2)	0.27026 (6)	0.0226 (5)	
C4	0.60627 (18)	0.3304 (2)	0.24887 (6)	0.0246 (5)	
H4A	0.6398	0.3897	0.2288	0.029*	
H4B	0.5596	0.2702	0.2333	0.029*	
C5	0.53483 (18)	0.4053 (2)	0.27790 (6)	0.0244 (5)	
C6	0.51360 (17)	0.3502 (2)	0.31886 (6)	0.0214 (5)	
C7	0.43583 (17)	0.4193 (2)	0.34753 (6)	0.0230 (5)	
H7	0.3668	0.4380	0.3321	0.028*	
C8	0.40825 (17)	0.3317 (2)	0.38289 (6)	0.0238 (5)	
C9	0.31381 (19)	0.3637 (2)	0.40908 (7)	0.0296 (5)	
C10	0.2868 (2)	0.2742 (2)	0.44399 (7)	0.0343 (6)	
H10A	0.2361	0.2078	0.4337	0.041*	
H10B	0.2481	0.3224	0.4658	0.041*	
C11	0.3870 (2)	0.2095 (2)	0.46289 (6)	0.0298 (6)	
C12	0.44987 (19)	0.1422 (2)	0.42801 (6)	0.0261 (5)	
H12A	0.5227	0.1156	0.4383	0.031*	
H12B	0.4094	0.0647	0.4198	0.031*	
C13	0.46462 (17)	0.2256 (2)	0.39124 (6)	0.0227 (5)	
C14	0.75809 (19)	0.1729 (2)	0.23874 (6)	0.0278 (5)	
H14A	0.8182	0.1276	0.2524	0.042*	
H14B	0.7878	0.2272	0.2169	0.042*	
H14C	0.7070	0.1112	0.2268	0.042*	
C15	0.78024 (19)	0.3455 (2)	0.29057 (7)	0.0295 (5)	
H15A	0.8387	0.2957	0.3035	0.044*	
H15B	0.7429	0.3968	0.3115	0.044*	
H15C	0.8119	0.4017	0.2696	0.044*	
C16	0.4605 (2)	0.3084 (2)	0.48427 (7)	0.0421 (7)	
H16A	0.4185	0.3519	0.5058	0.063*	
H16B	0.4859	0.3708	0.4640	0.063*	
H16C	0.5238	0.2656	0.4966	0.063*	
C17	0.3501 (2)	0.1099 (2)	0.49439 (7)	0.0395 (7)	
H17A	0.3046	0.0458	0.4807	0.059*	
H17B	0.3075	0.1514	0.5161	0.059*	
H17C	0.4146	0.0687	0.5065	0.059*	
C18	0.48347 (18)	0.5456 (2)	0.36303 (6)	0.0237 (5)	
C19	0.42695 (19)	0.6595 (2)	0.35885 (6)	0.0281 (5)	
H19	0.3565	0.6592	0.3464	0.034*	
C20	0.4708 (2)	0.7741 (2)	0.37241 (7)	0.0318 (6)	
H20	0.4305	0.8507	0.3689	0.038*	
C21	0.57294 (19)	0.7774 (2)	0.39100 (6)	0.0296 (5)	
H21	0.6043	0.8551	0.4002	0.035*	
C22	0.62764 (18)	0.6625 (2)	0.39566 (6)	0.0250 (5)	
C23	0.58551 (18)	0.5471 (2)	0.38256 (6)	0.0241 (5)	
H23	0.6252	0.4704	0.3868	0.029*	

### Atomic displacement parameters (Å^2^)

**Table:**

	*U*^11^	*U*^22^	*U*^33^	*U*^12^	*U*^13^	*U*^23^
O1	0.0265 (9)	0.0227 (9)	0.0221 (7)	0.0030 (7)	0.0026 (6)	0.0018 (6)
O2	0.0319 (10)	0.0312 (10)	0.0529 (10)	0.0085 (8)	0.0103 (8)	0.0031 (8)
O3	0.0332 (10)	0.0266 (10)	0.0319 (8)	0.0075 (8)	−0.0029 (7)	0.0042 (7)
O4	0.0405 (12)	0.0360 (12)	0.0655 (12)	−0.0072 (9)	−0.0202 (9)	0.0157 (9)
O5	0.0310 (10)	0.0320 (11)	0.0572 (11)	−0.0071 (8)	0.0037 (8)	−0.0187 (8)
N1	0.0283 (11)	0.0318 (12)	0.0297 (10)	−0.0046 (10)	0.0018 (8)	−0.0044 (8)
C1	0.0237 (12)	0.0211 (12)	0.0209 (10)	−0.0029 (10)	−0.0028 (8)	−0.0011 (8)
C2	0.0260 (12)	0.0187 (12)	0.0232 (10)	0.0014 (10)	−0.0016 (8)	−0.0011 (8)
C3	0.0248 (12)	0.0183 (12)	0.0246 (10)	−0.0003 (10)	0.0008 (8)	−0.0002 (8)
C4	0.0275 (12)	0.0234 (12)	0.0228 (10)	0.0001 (10)	0.0001 (9)	0.0000 (9)
C5	0.0220 (12)	0.0227 (13)	0.0286 (11)	−0.0012 (10)	−0.0045 (9)	−0.0003 (9)
C6	0.0214 (12)	0.0191 (12)	0.0236 (10)	−0.0002 (9)	−0.0016 (8)	−0.0018 (8)
C7	0.0198 (11)	0.0230 (12)	0.0261 (11)	0.0015 (10)	−0.0030 (8)	−0.0012 (9)
C8	0.0229 (12)	0.0224 (12)	0.0262 (10)	−0.0018 (10)	−0.0014 (9)	−0.0016 (9)
C9	0.0277 (13)	0.0249 (13)	0.0362 (12)	−0.0018 (11)	0.0035 (10)	−0.0045 (10)
C10	0.0342 (14)	0.0283 (14)	0.0403 (13)	−0.0020 (12)	0.0134 (10)	−0.0021 (10)
C11	0.0347 (14)	0.0253 (13)	0.0293 (11)	−0.0053 (11)	0.0079 (10)	−0.0030 (10)
C12	0.0272 (12)	0.0237 (13)	0.0273 (11)	−0.0023 (10)	0.0032 (9)	0.0004 (9)
C13	0.0194 (11)	0.0232 (13)	0.0253 (10)	−0.0035 (10)	0.0016 (8)	−0.0047 (9)
C14	0.0305 (13)	0.0264 (13)	0.0266 (11)	0.0005 (11)	0.0043 (9)	0.0008 (9)
C15	0.0302 (13)	0.0247 (13)	0.0336 (11)	−0.0027 (11)	−0.0029 (9)	−0.0019 (10)
C16	0.0536 (17)	0.0411 (17)	0.0318 (12)	−0.0137 (14)	0.0024 (11)	−0.0090 (11)
C17	0.0483 (17)	0.0366 (16)	0.0337 (12)	−0.0024 (13)	0.0133 (11)	0.0013 (11)
C18	0.0280 (12)	0.0224 (13)	0.0207 (10)	0.0004 (10)	0.0026 (8)	−0.0021 (8)
C19	0.0286 (13)	0.0261 (14)	0.0296 (11)	0.0041 (11)	−0.0028 (9)	0.0000 (9)
C20	0.0393 (15)	0.0213 (13)	0.0349 (12)	0.0077 (11)	0.0005 (10)	−0.0017 (10)
C21	0.0364 (14)	0.0240 (13)	0.0283 (11)	−0.0024 (11)	0.0042 (10)	−0.0028 (9)
C22	0.0255 (12)	0.0261 (13)	0.0236 (10)	−0.0030 (10)	0.0021 (8)	−0.0004 (8)
C23	0.0265 (12)	0.0211 (12)	0.0246 (10)	0.0021 (10)	0.0018 (9)	−0.0004 (9)

### Geometric parameters (Å, °)

**Table:**

O1—C1	1.378 (2)	C11—C17	1.531 (3)
O1—C13	1.381 (2)	C11—C16	1.538 (3)
O2—C9	1.230 (3)	C11—C12	1.540 (3)
O3—C5	1.230 (3)	C12—C13	1.492 (3)
O4—N1	1.227 (2)	C12—H12A	0.9900
O5—N1	1.231 (2)	C12—H12B	0.9900
N1—C22	1.472 (3)	C14—H14A	0.9800
C1—C6	1.340 (3)	C14—H14B	0.9800
C1—C2	1.488 (3)	C14—H14C	0.9800
C2—C3	1.533 (3)	C15—H15A	0.9800
C2—H2A	0.9900	C15—H15B	0.9800
C2—H2B	0.9900	C15—H15C	0.9800
C3—C14	1.531 (3)	C16—H16A	0.9800
C3—C4	1.532 (3)	C16—H16B	0.9800
C3—C15	1.533 (3)	C16—H16C	0.9800
C4—C5	1.507 (3)	C17—H17A	0.9800
C4—H4A	0.9900	C17—H17B	0.9800
C4—H4B	0.9900	C17—H17C	0.9800
C5—C6	1.474 (3)	C18—C19	1.389 (3)
C6—C7	1.515 (3)	C18—C23	1.397 (3)
C7—C8	1.510 (3)	C19—C20	1.389 (3)
C7—C18	1.534 (3)	C19—H19	0.9500
C7—H7	1.0000	C20—C21	1.385 (3)
C8—C13	1.338 (3)	C20—H20	0.9500
C8—C9	1.471 (3)	C21—C22	1.388 (3)
C9—C10	1.510 (3)	C21—H21	0.9500
C10—C11	1.528 (3)	C22—C23	1.385 (3)
C10—H10A	0.9900	C23—H23	0.9500
C10—H10B	0.9900		
			
C1—O1—C13	117.83 (17)	C17—C11—C12	108.93 (19)
O4—N1—O5	124.0 (2)	C16—C11—C12	110.6 (2)
O4—N1—C22	117.83 (19)	C13—C12—C11	112.29 (18)
O5—N1—C22	118.1 (2)	C13—C12—H12A	109.1
C6—C1—O1	123.00 (18)	C11—C12—H12A	109.1
C6—C1—C2	125.82 (18)	C13—C12—H12B	109.1
O1—C1—C2	111.17 (17)	C11—C12—H12B	109.1
C1—C2—C3	112.65 (18)	H12A—C12—H12B	107.9
C1—C2—H2A	109.1	C8—C13—O1	122.81 (19)
C3—C2—H2A	109.1	C8—C13—C12	126.15 (19)
C1—C2—H2B	109.1	O1—C13—C12	111.04 (18)
C3—C2—H2B	109.1	C3—C14—H14A	109.5
H2A—C2—H2B	107.8	C3—C14—H14B	109.5
C14—C3—C4	109.77 (17)	H14A—C14—H14B	109.5
C14—C3—C15	108.71 (18)	C3—C14—H14C	109.5
C4—C3—C15	110.81 (18)	H14A—C14—H14C	109.5
C14—C3—C2	110.01 (17)	H14B—C14—H14C	109.5
C4—C3—C2	107.53 (18)	C3—C15—H15A	109.5
C15—C3—C2	109.99 (17)	C3—C15—H15B	109.5
C5—C4—C3	113.96 (17)	H15A—C15—H15B	109.5
C5—C4—H4A	108.8	C3—C15—H15C	109.5
C3—C4—H4A	108.8	H15A—C15—H15C	109.5
C5—C4—H4B	108.8	H15B—C15—H15C	109.5
C3—C4—H4B	108.8	C11—C16—H16A	109.5
H4A—C4—H4B	107.7	C11—C16—H16B	109.5
O3—C5—C6	120.21 (19)	H16A—C16—H16B	109.5
O3—C5—C4	122.31 (19)	C11—C16—H16C	109.5
C6—C5—C4	117.45 (19)	H16A—C16—H16C	109.5
C1—C6—C5	118.54 (19)	H16B—C16—H16C	109.5
C1—C6—C7	123.00 (18)	C11—C17—H17A	109.5
C5—C6—C7	118.46 (18)	C11—C17—H17B	109.5
C8—C7—C6	108.39 (18)	H17A—C17—H17B	109.5
C8—C7—C18	111.21 (16)	C11—C17—H17C	109.5
C6—C7—C18	112.29 (18)	H17A—C17—H17C	109.5
C8—C7—H7	108.3	H17B—C17—H17C	109.5
C6—C7—H7	108.3	C19—C18—C23	118.5 (2)
C18—C7—H7	108.3	C19—C18—C7	121.75 (19)
C13—C8—C9	118.4 (2)	C23—C18—C7	119.76 (19)
C13—C8—C7	123.21 (19)	C18—C19—C20	121.7 (2)
C9—C8—C7	118.37 (19)	C18—C19—H19	119.2
O2—C9—C8	120.0 (2)	C20—C19—H19	119.2
O2—C9—C10	122.4 (2)	C21—C20—C19	120.4 (2)
C8—C9—C10	117.6 (2)	C21—C20—H20	119.8
C9—C10—C11	113.83 (19)	C19—C20—H20	119.8
C9—C10—H10A	108.8	C20—C21—C22	117.3 (2)
C11—C10—H10A	108.8	C20—C21—H21	121.4
C9—C10—H10B	108.8	C22—C21—H21	121.4
C11—C10—H10B	108.8	C23—C22—C21	123.4 (2)
H10A—C10—H10B	107.7	C23—C22—N1	118.3 (2)
C10—C11—C17	109.7 (2)	C21—C22—N1	118.3 (2)
C10—C11—C16	110.3 (2)	C22—C23—C18	118.7 (2)
C17—C11—C16	109.38 (19)	C22—C23—H23	120.7
C10—C11—C12	107.88 (18)	C18—C23—H23	120.7
			
C13—O1—C1—C6	6.2 (3)	C8—C9—C10—C11	32.6 (3)
C13—O1—C1—C2	−172.70 (17)	C9—C10—C11—C17	−173.52 (19)
C6—C1—C2—C3	22.0 (3)	C9—C10—C11—C16	65.9 (2)
O1—C1—C2—C3	−159.20 (17)	C9—C10—C11—C12	−55.0 (3)
C1—C2—C3—C14	−167.45 (17)	C10—C11—C12—C13	46.8 (2)
C1—C2—C3—C4	−47.9 (2)	C17—C11—C12—C13	165.81 (19)
C1—C2—C3—C15	72.8 (2)	C16—C11—C12—C13	−73.9 (2)
C14—C3—C4—C5	174.59 (18)	C9—C8—C13—O1	172.13 (18)
C15—C3—C4—C5	−65.3 (2)	C7—C8—C13—O1	−7.0 (3)
C2—C3—C4—C5	54.9 (2)	C9—C8—C13—C12	−7.8 (3)
C3—C4—C5—O3	148.0 (2)	C7—C8—C13—C12	173.1 (2)
C3—C4—C5—C6	−34.1 (3)	C1—O1—C13—C8	−4.3 (3)
O1—C1—C6—C5	−176.97 (18)	C1—O1—C13—C12	175.67 (17)
C2—C1—C6—C5	1.8 (3)	C11—C12—C13—C8	−17.4 (3)
O1—C1—C6—C7	3.2 (3)	C11—C12—C13—O1	162.64 (18)
C2—C1—C6—C7	−178.1 (2)	C8—C7—C18—C19	−112.2 (2)
O3—C5—C6—C1	−177.9 (2)	C6—C7—C18—C19	126.2 (2)
C4—C5—C6—C1	4.3 (3)	C8—C7—C18—C23	66.7 (2)
O3—C5—C6—C7	2.0 (3)	C6—C7—C18—C23	−54.9 (2)
C4—C5—C6—C7	−175.89 (18)	C23—C18—C19—C20	2.0 (3)
C1—C6—C7—C8	−12.4 (3)	C7—C18—C19—C20	−179.1 (2)
C5—C6—C7—C8	167.72 (18)	C18—C19—C20—C21	−0.6 (3)
C1—C6—C7—C18	110.8 (2)	C19—C20—C21—C22	−0.6 (3)
C5—C6—C7—C18	−69.0 (2)	C20—C21—C22—C23	0.4 (3)
C6—C7—C8—C13	14.3 (3)	C20—C21—C22—N1	−178.68 (18)
C18—C7—C8—C13	−109.6 (2)	O4—N1—C22—C23	−13.9 (3)
C6—C7—C8—C9	−164.80 (18)	O5—N1—C22—C23	166.23 (19)
C18—C7—C8—C9	71.3 (2)	O4—N1—C22—C21	165.2 (2)
C13—C8—C9—O2	−176.5 (2)	O5—N1—C22—C21	−14.7 (3)
C7—C8—C9—O2	2.6 (3)	C21—C22—C23—C18	1.0 (3)
C13—C8—C9—C10	0.2 (3)	N1—C22—C23—C18	−179.92 (17)
C7—C8—C9—C10	179.30 (19)	C19—C18—C23—C22	−2.2 (3)
O2—C9—C10—C11	−150.8 (2)	C7—C18—C23—C22	178.92 (18)
